# Synaptic integration by NG2 cells

**DOI:** 10.3389/fncel.2013.00255

**Published:** 2013-12-20

**Authors:** Wenjing Sun, Dirk Dietrich

**Affiliations:** Experimental Neurophysiology, Department of Neurosurgery, University Clinic BonnBonn, Germany

**Keywords:** NG2 cell, synapses, calcium signaling, dendrites, cable model

## Abstract

NG2 expressing oligodendrocyte precursor cells stand out from other types of glial cells by receiving classical synaptic contacts from many neurons. This unconventional form of signaling between neurons and glial cells enables NG2 cells to receive information about the activity of presynaptic neurons with high temporal and spatial precision and has been postulated to be involved in activity-dependent myelination. While this still unproven concept is generally compelling, how NG2 cells may integrate synaptic input has hardly been addressed to date. Here we review the biophysical characteristics of synaptic currents and membrane properties of NG2 cells and discuss their capabilities to perform complex temporal and spatial signal integration and how this may be important for activity-dependent myelination.

## Introduction

Oligodendrocyte precursor cells (OPCs) have received much attention in the recent past because they represent a large and enigmatic population of glial cells which exists throughout development and adulthood in all grey matter and white matter areas of the central nervous system (CNS; Nishiyama et al., 1999; Butt et al., 2005; Trotter et al., 2010). They are identified as non-vascular cells which strongly express the surface antigen NG2 and are frequently called NG2 cells. NG2 cells represent approximately 5% of all cells in the central nervous system (Dawson et al., 2003) and show a morphology which, at first glance, is related to that of microglial cells: their cell body is small, ~6–7 µm, and typically carries 5–7 delicate dendrites which branch irregularly and fill an approximately spherical volume spanning ~60 µm (Chittajallu et al., 2004; Lin and Bergles, 2004; Ge et al., 2006; Kukley et al., 2010). Around the late embryonic period NG2 cells start to colonise the forebrain from parenchymal progenitors and are found at a roughly constant density thereafter (Nishiyama et al., 1996; Kessaris et al., 2006). Remarkably, NG2 cells continue to proliferate even at later stages in life and are the largest population of cycling cells in the brain after the first postnatal week (Dawson et al., 2003). Currently, the only unequivocally confirmed function of NG2 cells is to replenish themselves and generate oligodendrocytes in healthy, diseased and aged CNS (Zhu et al., 2008; Kang et al., 2010; Tripathi et al., 2010; Young et al., 2013). However, NG2 cells persist in very large numbers even far beyond the main wave of oligodendrogenesis suggesting that there is still much to discover about additional roles of this type of glial cell. To underline this fact and emphasize that NG2 cells coexist with and are distinct from other types of glial cells throughout life, they have been identified as a fourth type of glial cell (Peters, 2004), termed polydendrocytes (Nishiyama et al., 2009), or synantocytes (Butt et al., 2005).

There are two remarkable features of NG2 cells which substantiate suspicion of additional functions for this abundant population of cells. Firstly, NG2 cells express a rich set of voltage gated ion channels (reviewed by Lin and Bergles, 2002). As in neurons, voltage gated sodium and potassium channels are easily identified in the voltage-clamped current pattern of NG2 cells (Chittajallu et al., 2004; Jabs et al., 2005; Karadottir et al., 2008; De Biase et al., 2010; Kukley et al., 2010; Clarke et al., 2012). However, NG2 cells do not possess an axon and do not fire action potentials (Bergles et al., 2000; Chittajallu et al., 2004; Lin et al., 2005; Ziskin et al., 2007; Mangin et al., 2008; Ge et al., 2009; Tong et al., 2009; De Biase et al., 2010, 2011, but see Karadottir et al., 2008), and therefore the purpose of the expressed voltage gated channels has remained obscure. Secondly and most remarkably, NG2 cells ubiquitously receive direct synaptic input from neighboring axons (Bergles et al., 2000; Lin and Bergles, 2004; Jabs et al., 2005; Lin et al., 2005; Ge et al., 2006; Kukley et al., 2007; Ziskin et al., 2007; Karadottir et al., 2008; Kukley et al., 2008; Ge et al., 2009; Velez-Fort et al., 2010). As reviewed elsewhere in more detail (Gallo et al., 2008; Bergles et al., 2010; Maldonado et al., 2011), this synaptic input is due to classical vesicular transmitter release at morphological sites of contact which do not appear to differ from classical neuronal synaptic junctions. In fact, synaptic currents recorded in NG2 cells are so similar to synaptic currents recorded in neurons that even experts have a hard time diagnosing the type of cell when judging the electrophysiological recording only. Evidence is growing that synaptic input to NG2 cells may be a mechanism to modify the development of oligodendrocytes by on-going neuronal activity. However, there are many ways that neuronal activity could influence the development of NG2 cells and a decisive experiment specifically assigning a functional role to quantal transmitter release at neuron-NG2 cell synapses has not yet been performed. Nevertheless, considering the biological efforts required to maintain this unconventional signaling it is likely that neuron-NG2 cell synapses are an important phenomenon: synapses on NG2 cells are observed in white and grey matter regions throughout life (Bergles et al., 2000; Chittajallu et al., 2004; Lin and Bergles, 2004; Jabs et al., 2005; Ge et al., 2006; Kukley et al., 2007; Ziskin et al., 2007; Karadottir et al., 2008; Kukley et al., 2008; Ge et al., 2009; De Biase et al., 2010; Velez-Fort et al., 2010) and in particular the assembly of synapses in white matter depends on long range transport of the components of the release machinery. They appear as early as NG2 cells are generated and have been demonstrated during the very first postnatal days of life (Kukley et al., 2008; Mangin et al., 2008; Velez-Fort et al., 2010). Neuron-NG2 cell synapses are surprisingly maintained when NG2 cells divide and are transferred to the daughter cells (Kukley et al., 2008; Ge et al., 2009) but are disassembled as soon as an NG2 cell differentiates (De Biase et al., 2010; Kukley et al., 2010). Finally, even adult-born NG2 cells participating in regenerating damaged white matter receive synaptic input after migrating from the subventricular zone to the site of injury (Etxeberria et al., 2010).

While there are many open questions about NG2 cells we focus this article on an important signaling step which we consider critical for understanding the ultimate functional role of neuron-NG2 cell synapses: The intriguing issue of how NG2 cells may integrate synaptic input without the possibility to generate an action potential or release transmitter from an axon. We specifically review fast and direct vesicular transmitter release from presynaptic neuronal specializations into a narrow cleft formed with the postsynaptic NG2 cell membrane. We do not consider so-called volume transmission or signals mediated by other ambient diffusible factors but this does not imply those signals may not be equally important.

## Synaptic input to NG2 cells

Besides glutamate and GABA receptors, O-2A progenitor cells or NG2 cells also express a large spectrum of other neurotransmitter receptors, such as nicotinic and muscarinic acetylcholine receptors, dopamine receptors, cannabinoid receptors, glycine receptors and purinergic receptors (Barres et al., 1990; Von Blankenfeld et al., 1991; Belachew et al., 1998; Bongarzone et al., 1998; Ragheb et al., 2001; Rogers et al., 2001; Molina-Holgado et al., 2002; Agresti et al., 2005; Velez-Fort et al., 2009). Since A2B5 positive O-2A progenitors also express NG2 (Stallcup and Beasley, 1987), they may be considered the *in vitro* equivalents of native NG2 cells. Later studies employing patch-clamp recordings of NG2 cells in brain slices have also identified NMDA- and kainate receptors (Kukley and Dietrich, 2009; De Biase et al., 2010). Here, we focus on glutamate and GABA receptors as direct synaptic release onto NG2 cells has been described only for these two transmitters (Gallo et al., 2008). Under physiological conditions synaptic release of glutamate and GABA has only been shown to activate AMPA-type and GABA-A receptors as the postsynaptic responses in NG2 cells are completely blocked by the respective specific antagonists (Bergles et al., 2000; Lin and Bergles, 2004; Jabs et al., 2005; Ge et al., 2006; Kukley et al., 2007, 2008, 2010; Velez-Fort et al., 2010).

Bergles et al. (2000) first reported quantal glutamatergic transmission from CA3 pyramidal neurons onto NG2 cells in rat hippocampus (Bergles et al., 2000). Following this discovery, multiple other studies demonstrated that NG2 cells receive both glutamatergic and GABAergic inputs from neurons in both grey and white matter of different brain regions, including cerebral cortex, hippocampus, cerebellum, corpus callosum and optic nerves (Bergles et al., 2000; Lin and Bergles, 2004; Jabs et al., 2005; Lin et al., 2005; Ge et al., 2006; Kukley et al., 2007; Ziskin et al., 2007; Karadottir et al., 2008; Kukley et al., 2008; Mangin et al., 2008; Ge et al., 2009; Velez-Fort et al., 2010).

The modes and possibilities of integration of synaptic input by NG2 cells are critically influenced by the biophysical properties of the postsynaptic conductance changes mediated by neurotransmitter receptor activation. Thanks to the many detailed electrophysiological studies on neuron-NG2 cell synapses, we now have a fairly good quantitative understanding of the synaptically induced changes of membrane conductance. We first review this quantitative data on synaptic conductance changes in NG2 cells in terms of current amplitudes measured with patch-clamp recordings. We then use this data in the subsequent sections to discuss how these conductance changes could be integrated by membrane potential or intracellular ion concentration.

As mentioned above glutamatergic synaptic currents in NG2 cells are predominantly mediated by AMPA receptors and therefore display fast kinetics. Quantal currents, currents in response to the release of an individual transmitter-filled vesicle, rise to peak within ~1 ms, decay with a time constant of ~1.5 ms and peak at approximately 10 pA (Bergles et al., 2000; Lin et al., 2005; Kukley et al., 2007; De Biase et al., 2010; Kukley et al., 2010). The current amplitude shows a linear dependence on the membrane voltage up to the reversal potential of approximately 0 mV (using standard internal and external ion concentrations). NG2 cells can receive up to 100 glutamatergic synaptic connections from neighboring axons (Kukley et al., 2007). Thus, even if we assume a relatively low release probability (*P_r_*) of 25% (Kukley et al., 2007), simultaneous firing of presynaptic neurons can be expected to produce a postsynaptic current of at least 250 pA in an average NG2 cell. Taken together, with its rapid kinetics and large driving force glutamatergic synaptic input can reliably transmit firing frequencies of presynaptic neurons up to 100 Hz and also precisely encode the number of firing neurons over a large range.

Synaptic currents due to vesicular release of GABA are primarily mediated by chloride-selective GABA-A receptors. In contrast to mature neurons, GABA-ergic synaptic input depolarizes NG2 cells as the chloride reversal potential is approximately −40 mV (Lin and Bergles, 2004; Passlick et al., 2013). Therefore, GABA is not an electrically inhibitory neurotransmitter for NG2 cells and, around their resting membrane potential, these cells cannot differentiate between GABA-ergic and glutamatergic synaptic input based on the direction of the voltage change. Due to the asymmetric distribution of chloride ions across the cell membrane, the voltage dependence of GABA-ergic currents shows Goldman rectification (Lin and Bergles, 2004; Kukley et al., 2008): upon depolarization the currents decrease over-proportionally with the decreasing driving force and, as outward currents, increase over-proportionally beyond the reversal potential. Considering the different recording conditions of previous studies, the average GABA-A receptor mediated quantal current amplitude would be approximately 10 pA under unperturbed conditions. The amplitude and the rise time (approximately 3 ms) of GABA-ergic currents are thus comparable to those of glutamatergic synaptic currents. However, the GABA-ergic currents are much more long-lasting and decay with a time constant of approximately 30 ms (Lin and Bergles, 2004; Jabs et al., 2005; Kukley et al., 2008; Velez-Fort et al., 2010). The number of GABA-ergic synaptic contacts on NG2 cells has not yet been determined. But based on the observation that the frequency of miniature and spontaneous GABA-ergic synaptic currents is much lower than the frequency of glutamatergic currents under identical conditions (Jabs et al., 2005; Kukley et al., 2008) it appears likely that the number of GABA containing synapses is at least five-fold smaller. Using 50% as an estimate of the release probability at a typical inhibitory synapses in the hippocampus (Kraushaar and Jonas, 2000) in the absence of a specific estimate for NG2 cells, these numbers predict a maximal postsynaptic GABA-ergic current of 100 pA. In summary, because of its slow time course and limited driving force, GABA-ergic synaptic input is not well suited to precisely transmit presynaptic firing frequency or number of active neurons. Its importance may rather be to translate low-frequency firing of individual or a small number of axons into modulatory changes of the resting potential of NG2 cells.

The above-mentioned characteristics of synaptic input to NG2 cells have been determined in the second postnatal week. In the third and fourth postnatal week, when myelination peaks in many areas of the brain, the electrical impact of transmitter release conspicuously increases. The amplitude of glutamatergic and GABA-ergic synaptic currents in NG2 cells increases 1.5–2 fold (Lin and Bergles, 2004; Mangin et al., 2008; Velez-Fort et al., 2010). For glutamate-mediated currents this increase is likely due to a stronger unitary connection between neurons and NG2 cells (Mangin et al., 2008). The mechanism for the amplitude increase of GABA-ergic currents is less clear because it is accompanied by a significant slowing of the rise and decay times of the currents. The authors propose that the diffusional distance between GABA-ergic synaptic terminals and NG2 cells in neocortex increases with the age of the animal and causes the slower kinetics (Velez-Fort et al., 2010; Maldonado et al., 2011). Yet, if this is the case it remains unclear why the amplitude is larger as it would be expected to be reduced by the larger diffusion distance. Another study analyzing hippocampal NG2 cells did not observe different kinetics of GABA-ergic synaptic currents between NG2 cells in postnatal vs. adult tissue (Lin and Bergles, 2004). It is important to keep in mind that the described developmental strengthening of synaptic input to NG2 cells existing within an environment with highly active on-going myelination is different from what happens if an individual NG2 cell actually differentiates. NG2 cells which differentiate into pre-myelinating oligodendrocytes stop expressing NG2 and gradually loose synaptic input (De Biase et al., 2010; Kukley et al., 2010).

## Integration of synaptic input by membrane potential

Electrical integration of synaptic input by temporal changes of the membrane potential represents the classical mode of synaptic integration by neurons. Spatial and temporal summation of primarily dendritic synaptic input at the initial axon segment is converted to a single or a train of action potentials. NG2 cells do not have an axon nor do they fire action potentials. Nevertheless, synaptically induced changes in membrane potential may still be important by modulating the intracellular calcium concentration or electrogenic transport processes in NG2 cells (see below).

The passive electrical properties of NG2 cells determine the electrical impact of the relatively small synaptic currents described above: a hyperpolarized resting membrane potential between −80 and −90 mV provides a large driving force for glutamatergic and GABA-ergic currents and an input resistance of approximately 400 MΩ, together with a fast membrane time constant of 5–10 ms, allow even the brief synaptic conductance change of glutamate receptors to induce pronounced depolarizations (Bergles et al., 2000; Jabs et al., 2005; Lin et al., 2005; Ziskin et al., 2007; Mangin et al., 2008; Haberlandt et al., 2011). Thus, the above-mentioned maximal synaptic glutamate current of 250 pA can be predicted to depolarize a passive NG2 cell lacking any voltage-gated ion channels by approximately 60 mV when assuming that temporal filtering and the decreasing driving force during the voltage excursion reduce the peak voltage response by approximately 40%. The maximum depolarization of a passive NG2 cell caused by a compound GABA-ergic postsynaptic potential may reach approximately 25 mV when also correcting the peak amplitude for an attenuation of approximately 40% due to stronger Goldman rectification and weaker temporal filtering compared to the glutamatergic input. When considering repetitive synaptic input, as occurs during trains of presynaptic action potentials, cumulative synaptic depolarization of NG2 cells may be substantial and reach the reversal potentials of synaptic neurotransmitter receptors as proposed by Jabs et al. (2005).

The existence of voltage-gated ion channels in NG2 cells was first reported for cultured O-2A progenitor cells (Sontheimer et al., 1989; Barres et al., 1990; Gallo et al., 1996; Williamson et al., 1997) and later confirmed by patch-clamp recordings of progenitors in brain slices. First confirmation of voltage gated channels came from recordings of so called complex glial precursor cells (Steinhauser et al., 1994a). The term “complex glial precursor cells” was used before the concept of NG2 cells and oligodendrocyte precursor cells had been established. Retrospectively, however, it seems safe to classify the cells recorded at that time as at least a subpopulation of NG2 cells (Jabs et al., 2005). At the present time, a number of patch clamp studies confirm the existence of voltage-gated ion channels in NG2 cells recorded in acute brain slices as reviewed by Lin and Bergles (2002). Upon depolarizing voltage steps to approximately −40 mV, prominent outward currents in the range of several nA are observed during patch-clamp recordings of NG2 cells. Thus, it is clear that the voltage response of NG2 cells to synchronous activity of presynaptic neurons will not only be determined by the passive membrane properties but will likely be significantly modulated by voltage-activated ion channels. The recruitment of voltage-gated channels in NG2 cells by synaptic input has not yet been experimentally addressed. Nevertheless, existing voltage-clamp data allow us to outline possible scenarios and raise important key questions about electrical synaptic integration in NG2 cells.

Because of their pronounced amplitude (measured by voltage steps to 0 mV) three families of ion channels are likely to be most important for electrical integration of synaptic input: A-type potassium channels (1–2 nA), delayed-rectifier (DR-type) potassium channels (0.5–1.5 nA) and fast voltage-activated sodium channels (50–400 pA) (Steinhauser et al., 1994b; Kressin et al., 1995; Yuan et al., 2002; Chittajallu et al., 2004; Jabs et al., 2005; Xie et al., 2007; De Biase et al., 2010; Kukley et al., 2010). Although voltage-activated calcium channels and persistent sodium channels have also been described in NG2 cells, their direct effect on synaptic potentials is probably negligible because at physiological ion concentrations their amplitude ranges below approximately 50 pA (Akopian et al., 1996; Tong et al., 2009; Haberlandt et al., 2011). In contrast, the observed amplitudes of A-type, DR-type and sodium channels clearly outcompete the maximal synaptic current amplitudes and are therefore well suited to strongly modulate or counteract synaptic depolarizations.

However, whether synaptic potentials recruit voltage-gated channels also depends on the gating kinetics and voltage range of activation of the channels. A detailed analysis of these parameters is provided by the pioneering work of Steinhauser et al. (1994b) on the above mentioned complex precursor cells in hippocampal slices (Steinhauser et al., 1994b). It was found that A-type potassium channels are activated around −40 mV, rapidly open with a time constant of 0.5–1.5 ms, and deactivate with a time constant of 10–15 ms. In contrast, DR-type potassium channels slowly activate with a time constant of approximately 10 ms when the membrane potential is depolarized above −20 mV and hardly inactivate. Finally, as is known from neurons, voltage-activated sodium channels open very fast, in less than 0.5 ms, and also inactivate with a time constant around 2 ms. Interestingly, the activation threshold for voltage activated sodium channels is −40 mV, comparable to the threshold of A-type potassium channels. How do these kinetic parameters and voltage ranges relate to synaptic input?

When comparing the gating properties of DR-type channels to the kinetics and amplitudes of the above described synaptic conductance changes it seems clear that glutamatergic or GABA-ergic synaptic potentials can hardly recruit this type of potassium channel because it activates too slowly. On the other hand, A-type potassium channels activate sufficiently fast to modulate synaptic potentials. It seems reasonable to assume that glutamatergic synaptic potentials in response to synchronous action potentials in many presynaptic neurons are reduced in amplitude or shortened by an enhanced repolarization through A-type channels. Such shaping of glutamatergic potentials could be frequency-dependent for repetitive release events because A-type potassium channels rapidly inactivate and are therefore likely unable to maintain this modulation at higher frequencies. In other words, higher frequencies of presynaptic firing will not be attenuated and A-type channels may thus provide a mechanism of frequency detection. GABA-ergic synaptic potentials are much less likely to activate A-type potassium channels as the chloride reversal potential does not allow the membrane to be depolarized beyond the activation threshold for this type of channel. Nevertheless, since A-type potassium channels readily inactivate with even small depolarizations from the resting potential (Steinhauser et al., 1994b), GABA-ergic potentials may functionally eliminate A-type channels and pave the way for a more pronounced depolarization by glutamatergic potentials, thus functioning as a detector of coincident activity of several presynaptic axons.

**Figure 1:**
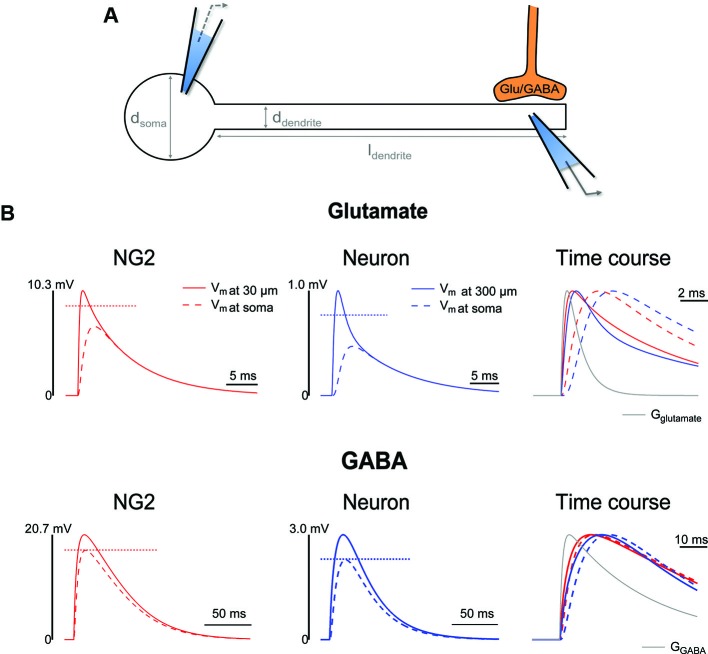
**Comparison of dendritic filtering between neuron and NG2 cell models. (A)** Scheme of the “ball and stick” model used to study the propagation of synaptic potentials along the dendrite. The “ball and stick” model represents a soma connected to a single dendrite. At the end of the dendrite we connected a synapse (orange) which caused a temporary conductance change. The time course of the conductance was modeled with a double exponential function to match recordings of miniature synaptic currents. The glutamatergic conductance change was simulated with τ_rise_ = 0.25 ms and τ_decay_ = 1 ms, the GABA-ergic conductance change was simulated with τ_rise_ = 1 ms and τ_decay_ = 30 ms. **(B)** Upper row: simulation of dendritic filtering for glutamatergic synaptic inputs along dendrites. The dendritic voltage response (continuous lines) in NG2 cells (red lines, left panel) is substantially larger than in neurons (blue lines, middle panel) owing to the smaller *d*_dendrite_ and therefore larger input resistance in the glial cell model. In both types of cells there is a pronounced attenuation of the peak of the synaptic potential to ~65% and 45%. Note that the reduction of the peak of the synaptic potential is much larger than the expected steady state attenuation in this model (indicated by the horizontal dotted lines. The right panel displays the normalized voltage responses along with the synaptic conductance change (*G*_glutamate_, grey line) to demonstrate the distinct time courses. Note the strong filtering of the voltage response during propagation from the end of the dendrite to the soma: Whereas the dendritic synaptic potential peak very soon after the peak of the conductance chance, the somatic potentials reach their peak only ~3–4 ms later. Lower row: as above but on a different time scale for GABA-ergic synaptic inputs. Due to the much slower kinetics of the GABA-ergic synaptic conductance change, the peak attenuation is well predicted by steady state properties of the model. Also, temporal filtering during propagation is negligible: while there is a clear delay of the peak of the voltage response in the dendrite when compared to the synaptic conductance change (*G*_GABA_, grey line), the voltage waveform is hardly altered by the spread to the soma. The reversal potentials were 0 mV and −43 mV for glutamate and GABA receptors. The amplitude of the synaptic conductance change was chosen to match (somatic) whole cell voltage-clamp experiments: the amplitude was adjusted such that a perfect dendritic voltage clamp recording registered a peak amplitude of 10 pA for both types of transmitters and for both types of cells. It is important to note that the model used follows a very simplistic view. Real cells have many more dendrites and each dendrite may branch several times. Omitting all but one dendrite causes the dendrites and the cell somata to show a greater input resistance which lets the dendritic voltage responses appear larger than in a real cell. However, while the absolute voltages should be treated with care, the comparison between cell types and between dendrite and soma are still valid.

When a neuron is depolarized above a certain threshold, fast voltage-activated sodium channels are known to cause regenerative spike-like depolarizations, called action potentials. Action potentials are the primary result of synaptic integration in neurons and are defined by characteristic properties: (1) All or none behavior: if the cell is depolarized above a relatively sharp threshold, it fires a full-blown action potential and it remains silent if the depolarization is too small; (2) Neuronal action potentials are brief and fast: they typically last 1–2 ms and their amplitude clearly is overshooting (reaching the positive voltage range); and (3) Amplitude and kinetics of the action potential are stereotyped: over a large range of frequencies, action potentials can be elicited without a decrement in the amplitude or a strong broadening of the shape. In particular due to their all-or-none behavior action potentials radically affect the integration of synaptic input. Below threshold each individual synaptic potential contributes to the size of the electrical response whereas the number of active synapses is of little importance once accumulated synaptic input reaches the action potential threshold. Further, the amplitude and the speed of action potentials are substantially larger than that of synaptic potentials themselves and due to this, action potentials very effectively open a number of voltage-gated ion channels such as calcium channels. It is therefore a major question whether NG2 cells are able to fire action potentials. There is a single frequently cited publication reporting the occurrence of action potentials in a subpopulation of NG2 cells in cerebellar white matter (Karadottir et al., 2008). Cerebellar white matter is bounded by granule cells and can be very thin inside the base of two neighboring folia. Action potentials of some granule cells in postnatal cerebellar grey matter (the developmental period studied by Karadottir et al., 2008) very closely resemble the action potentials reported for cerebellar white matter NG2 cells (compare Figures 1B, 2B in D’angelo et al., 1997 to Figure 4A, B in Karadottir et al., 2008). Unfortunately, Karadottir et al. (2008) neither showed nor mentioned NG2 immunoreactivity or the morphology of the spiking cells so it cannot be irrevocably ruled out that some granule cells might have possibly been mistaken for NG2 cells. When using NG2-DsRed mice to identify NG2 cells in cerebellar white matter at postnatal day 7–8 in our laboratory, we did not observe action potentials (*n* = 6, Kukley and Dietrich, unpublished observation) and the current clamp responses obtained were well in line with what is known from NG2 cells (Lin and Bergles, 2002). A number of other publications analyzing current clamp recordings of NG2 cells in different brain regions of rats and mice also did not observe action potentials in NG2 cells (Bergles et al., 2000; Chittajallu et al., 2004; Lin and Bergles, 2004; Lin et al., 2005; Ziskin et al., 2007; Mangin et al., 2008; Ge et al., 2009; Tong et al., 2009; De Biase et al., 2010, 2011). Nevertheless, it is clear that NG2 cells express a variable amount of voltage-activated sodium channels (Steinhauser et al., 1992; Gallo et al., 1996; Bergles et al., 2000; Diers-Fenger et al., 2001; Chittajallu et al., 2004; Lin and Bergles, 2004; Ge et al., 2006; Karadottir et al., 2008; Kukley et al., 2008; Ge et al., 2009; De Biase et al., 2010; Kukley et al., 2010; Clarke et al., 2012). Even if these sodium channels do not generate action potentials they still can be very important in amplifying synaptic input. To date it has not yet been tested whether synaptic depolarizations are amplified by sodium channels but some insight into the role of sodium channels can be obtained by analyzing the response to rectangular shaped current injections typically used to characterize NG2 cells in whole-cell current clamp recordings. Many investigators report that upon stronger current injections NG2 cells generate additional bump-like depolarizations which ride on the exponentially relaxing membrane potential. These bump-like voltage excursions occur with a delay of > 10 ms to the onset of the current injection, their amplitude rarely exceeds 10 mV and they typically last longer than 20 ms and thereby do not fulfil any of the abovementioned criteria for action potentials (Chittajallu et al., 2004; Mangin et al., 2008; Ge et al., 2009; Tong et al., 2009; De Biase et al., 2010; Clarke et al., 2012). It is likely that voltage-activated sodium channels underlie these bump-like events and also amplify and/or accelerate the rising phase of synaptic potentials in NG2 cells. In this way sodium channels may emphasize certain larger synaptic events and facilitate their discrimination against background activity. Whether sodium channels can activate quickly enough to enhance a synaptic input before the large amplitude A-type current counteracts the depolarization remains to be tested and will depend largely on the exact kinetics, amplitudes, and membrane time constants involved.

Neuron-NG2 cell synapses possess a fully competent release machinery which is capable of repetitively liberating transmitter in response to trains of presynaptic action potentials causing barrages of synaptic potentials in NG2 cells (Kukley et al., 2007). While, as described above, A-type potassium channels and fast sodium channels can be expected to rapidly inactivate during such barrages, DR-type potassium channels are ideally suited to countervail this repetitive synaptic activity. They have sufficient time to activate and remain activated as long as the activity is going on. In certain brain regions NG2 cells *in vivo* may indeed experience strong and enduring synaptic input and it may be postulated that it is the role of DR-type channels to set the steady state or plateau membrane potential under such conditions. As the frequency of presynaptic input determines the temporal summation of synaptic potentials and thereby the depolarization of NG2 cells, the voltage dependence of DR-type channels likely determines the frequency-response curve of NG2 cells and may shape it to display a resonance behavior such as that known for M-type potassium channels in hippocampal neurons (Hu et al., 2002; Peters et al., 2005).

Those NG2 cells which persist into adulthood show a strong trend to down regulate voltage-gated ion channels and to increase the expression of un-gated leak channels, lowering the cells’ input resistance (Kressin et al., 1995; Maldonado et al., 2013). As mentioned above, in the same developmental period the amplitude of synaptic currents increases. It is likely that the increase in synaptic input for a certain period can compensate the drop of the cell’s input resistance. However, eventually the expression of background potassium channels (leak channels) will short-circuit the depolarization from synaptic input. Therefore, starting from approximately the sixth postnatal week, shaping of synaptic potentials by voltage-gated channels becomes much less likely; synaptic depolarizations may not reach the threshold for activating voltage-gated channels and the density of voltage-gated channels probably is also too low to substantially impact the membrane potential of NG2 cells.

Notably, NG2 cells which are just transitioning to pre-myelinating oligodendrocytes down regulate leak channels and continue to show voltage-activated channels and thus are more receptive to synaptic input. As this happens during a developmental period during which there is a general increase of synaptic transmission onto NG2 cells, it is likely that there is a brief window in the development of an individual NG2 cell when it experiences pronounced synaptic depolarization and shaping by voltage-gated ion channels. It is worth mentioning that the developmental change in ion channel expression by differentiating NG2 cells has also been observed for differentiating A2B5 + O-2A progenitor cells *in vitro* (Sontheimer et al., 1989; Barres et al., 1990). The strong electrophysiological and morphological similarities between oligodendroglial precursor cells *in vitro* and NG2 cells in situ raises the question whether OPCs co-cultured with neurons may also form functional synaptic connections. Such co-cultures could be an excellent model to study cell biological roles of individual neuron-NG2 cell synapses on time frames which are not compatible with slice experiments (> 8 h).

It should be kept in mind that our predictions regarding recruitment of voltage gated channels by synaptic potentials in NG2 cells need experimental verification as the interplay of the exact membrane and gating time constants as well as voltage dependencies within an individual cell can crucially change the outcome. Nevertheless, we expect that it is the main purpose of voltage gated channels to shape synaptic potentials and it will be interesting to see whether the purpose of this shaping is to dampen or enhance synaptic input in a frequency-independent manner or to allow NG2 cells to distinguish glutamatergic from GABA-ergic transmission, both of which are depolarizing.

## Dendritic integration of synaptic input by local membrane potential

NG2 cells display an elaborate tree of dendrites giving rise to a total surface area of approximately 2000 µm^2^ as assessed by a 3-D reconstruction of a confocal dataset (Kukley et al., 2007). If we assume the diameter and shape of the soma of NG2 cells to be a 6.5 µm sphere, it follows that the dendritic tree of NG2 cells provides more than 90% of the surface area. It is therefore likely that the vast majority of neuron-NG2 cell synapses are formed on dendrites of the glial cell. This is qualitatively consistent with electron micrographs demonstrating synaptic contacts on NG2 cell dendrites (Lin and Bergles, 2004; Kukley et al., 2007; Ziskin et al., 2007; Kukley et al., 2008; Haberlandt et al., 2011). Thus, the question arises whether NG2 cells are capable of local integration of synaptic input in dendrites. Considering that the average length of an individual dendrite leaving the NG2 cell soma is only approximately 30 µm (derived from the average area covered by the dendritic tree of NG2 cells in a 2D projection, Kukley et al., 2010) it may be assumed that NG2 cells are electrically compact and synaptic potentials well propagate throughout the dendritic tree prohibiting local signaling. On the other hand, electron micrographs demonstrate that NG2 cell dendrites receiving synapses are exquisitely thin and show diameters of 200 nm or even less (Kukley et al., 2007; Haberlandt et al., 2011) which may favor voltage attenuation and local signaling.

To date, local integration in or electrical properties of dendrites of NG2 cells have not been determined. To obtain a basic understanding of possible dendritic integration in NG2 cells we extended the above biophysical estimates and simulated the spread of glutamatergic and GABA-ergic synaptic potentials with a simple “ball and stick” cable model in the NEURON simulation environment (Hines and Carnevale, 1997). To set up such a simulation one needs to choose values for the specific membrane capacitance, *C*_m_, the specific axial/cytoplasmic resistance, *R*_a_, and the specific membrane resistance, *R*_m_. We assume *C*_m_ and *R*_a_ are comparable to neurons and use values which have been established in neuronal models (1 µF/cm^2^, 100 Ω/cm, Gentet et al., 2000; Magee and Cook, 2000). The specific membrane resistance depends on the density of background potassium channels and can vary across cell types. We use the above estimate of the total surface area of NG2 cells (~2000 µm^2^, Kukley et al., 2007) and an average input resistance of NG2 cells from the literature (400 MΩ, see above) and calculate *R*_m_ to be approximately 8000 Ω cm^2^ (= 400 MΩ × 2000 µm^2^). According to infinite cable model, *R*_m_ and *R*_a_ together with the dendrite diameter, *d* = 0.2 µm, result in a space constant, *λ*, of 200 µm for NG2 cell dendrites (λ=Rmd4Ra, Rall, 1969). In contrast, a typically much larger primary neuronal dendrite, *d* = 1.5 µm (Routh et al., 2009), with *R*_m_ of approximately 10000 Ω cm^2^ (Magee and Cook, 2000) shows a *λ* of approximately 610 µm. The space constant *λ* states the distance from the site of a steady state voltage deflection at which the voltage has decayed to 1/e (~37%), meaning that a larger *λ* implies a weaker attenuation along the dendrite of a neuron when compared to an NG2 cell. (During revision of this manuscript a first study appeared which experimentally derived a passive cable model for NG2 cells and supports the basic conclusions drawn here Chan et al., 2013).

However, the space constant derived from an infinite cable model is not very meaningful for describing the spread and attenuation of synaptic potentials in a real cell because this model does not account for the significant leak caused by the somatic conductance and because the rapid synaptic conductance changes do not allow the membrane potential to reach steady-state. To account for these effects, we represented NG2 cells and CA1 neurons with the above-mentioned simplistic “ball and stick” model (Figure 1A). For NG2 cells we attached a 30 µm long dendrite (*d* = 0.2 µm) to a 6.5 µm wide spherical soma. For CA1 neurons we connected a 300 µm dendrite (*d* = 1.5 µm) to a soma with a diameter of 18 µm (Losonczy and Magee, 2006; Wierenga et al., 2008; Routh et al., 2009). For each type of cell, we used the specific membrane parameters introduced above and attached a single synapse at the end of the dendrites, i.e. at 30 and 300 µm. To study the dendritic voltage propagation and compare filtering between cell types, we simulated quantal glutamatergic and GABA-ergic synaptic conductance changes whose kinetics and amplitudes were derived from recordings of miniature synaptic currents in NG2 cells as introduced above (Figure 1B, see figure legend for details). For both, the NG2 cell (red) and the neuron (blue), we recorded the voltage (see recording electrodes in scheme) directly beneath the synapse (continuous line) and at the soma (dashed line). A striking difference between the two cell types is the substantially larger depolarization of NG2 cell dendrites (factor of 6–10), despite the same synaptic conductance change, which is due to the higher dendritic input resistance in NG2 cells. However, attenuation of the peak and temporal filtering of synaptic input is remarkably similar between NG2 cells and neurons. Attenuation and filtering is strong for the rapid conductance change of glutamatergic events; upon propagation from the end of the dendrite to the soma the amplitude of synaptic potentials is decreased to approximately 45–65% and the peak amplitudes occur significantly delayed at the soma (compare the full lines to the dashed lines in the rightmost panel). Attenuation and filtering of the GABA-ergic events, on the other hand, is weak for both types of cells; due to the slow time course of the GABA-ergic conductance change, the peak amplitude of the synaptic potential is only reduced to approximately 75–80% which almost equals the attenuation of a steady-state voltage change (indicated by the horizontal dotted lines). Furthermore, temporal filtering is negligible when comparing the time courses of the dendritic and somatic voltage waveforms in neurons and NG2 cells (rightmost panel). Taken together, whereas the small geometry of NG2 cell dendrites leads to a much more pronounced filtering and attenuation along dendrites in absolute terms, dendritic voltage propagation is very comparable to neurons when corrected for the typically much shorter dendrites of NG2 cells (i.e. 30 vs. 300 µm maximal length). Therefore, in terms of electronic compactness, NG2 cells may be considered a miniature edition of neurons, fully capable of downscaled dendritic computation.

Dendritic computation may be an advantage or even necessary for NG2 cells if transmitter release from a neighboring axon plays a role for myelination of the latter. While there is currently no data to directly demonstrate that neuron-NG2 cell synapses indeed are involved in selecting axons for myelination or in initiating or delaying the process of enwrapping or myelination, a number of studies indirectly support this concept as reviewed elsewhere (Fields, 2008). In this concept there is a double need for local synaptic integration by NG2 cells: firstly, as known from synaptic integration by neurons, synaptic signaling must in some way be sufficiently local such that the NG2 cell can identify and selectively respond to the synaptic activity of an individual axon. Secondly, and in contrast to neurons which generate a global output in the form of an action potential, NG2 cells need to produce a local response in the dendrite which specifically interacts with the axon which gave rise to the synaptic input. Such interaction may for example involve in initiating enwrapping or repelling the axon.

A recent paper reports local protein synthesis and differential calcium signaling in dendrites vs. the soma of NG2 cells in a cultured OPC-neuron preparation (Wake et al., 2011). At first glance, this paper seems to lend strong support to the idea of dendritic computation. However, care should be taken to equate the findings of Wake et al. (2011) with the concept of local integration of synaptic input as described above. Firstly, it is unlikely that transmission at direct neuron-NG2 cell synapses is underlying the phenomenon observed by Wake et al. (2011). The authors experimentally interfered with all neuronal synapses and not specifically with axo-glial synapses and the pharmacological profile of the phenomenon is not consistent with that of neuron-NG2 cell synapses. Further, the phenomenon was blocked by inhibiting NMDA and metabotropic glutamate receptors but not by blocking AMPA receptors while the converse would be expected for direct axon-NG2 cell synapses (see above). Secondly, the distinct calcium signaling in dendrites vs. the soma may well be due to a differential distribution of the involved receptor family on the NG2 cell membrane rather than due to a limited intracellular spread of the electrical signal. Nevertheless, the study by Wake et al. (2011) is an important contribution in many respects; in particular by substantiating the idea of activity-dependent myelination and by showing differential integration of signals from glutamate- vs. ATP-gated receptors.

## Integration of synaptic input by intracellular ion concentration

In this section we focus on changes in the intracellular ion concentration which result from activation of neurotransmitter receptors by vesicular release at neuron-NG2 cell synapses. As NG2 cells cannot convert synaptic depolarizations into action potential firing, changes in intracellular ion concentration directly or indirectly associated with neurotransmitter receptor opening may be an alternative way to initiate further cellular signaling.

### Intracellular calcium concentration

The depolarization caused by synaptic input may directly open voltage-gated calcium channels. The expression of both high voltage-activated (HVA) and low voltage-activated (LVA) calcium channels has been demonstrated in cultured O-2A progenitor cells (Williamson et al., 1997) and in complex glial precursor cells in hippocampal and corpus callosum slices (Berger et al., 1992; Akopian et al., 1996). These calcium channels were found to activate even with modest depolarizations to −60 to −40 mV. Further, but less compelling, support for functional voltage-activated calcium channels in NG2 cells is provided by studies which observed increases in intracellular calcium concentration (imaging) upon bath-application of neurotransmitters or a high potassium concentration which was prevented by antagonists of voltage-gated calcium channels or by employing voltage-clamp mode (Kirchhoff and Kettenmann, 1992; Kirischuk et al., 1995; Tanaka et al., 2009; Tong et al., 2009; Paez et al., 2010; Haberlandt et al., 2011). The existence of voltage-gated calcium channels is also supported by a recent single cell RT-PCR study revealing expression of L-type and T-type channels in hippocampal NG2 cells (Haberlandt et al., 2011). Calcium channel proteins have so far not been directly demonstrated in NG2 cells but Chen et al. (2000) observed a conspicuous increase in immunoreactivity against R-type channels in white matter tracts just before the onset of myelination (Chen et al., 2000).

Recalling that the passive properties of NG2 cells may allow for a pronounced summation of synaptic potentials, it is conceivable that GABA-ergic and glutamatergic transmission generate increases in intracellular calcium by activating voltage-gated calcium channels. Two studies tested for this possibility: Velez-Fort et al. (2010) did not observe calcium signals in NG2 cells in bulk-loaded slices by trains of extracellular stimulation (see supplementary information in Velez-Fort et al., 2010). Unfortunately, their experimental paradigm did not allow verification that the extracellular stimulation indeed activated synaptic inputs to the optically recorded NG2 cells. Haberlandt et al. (2011) used a similar stimulation paradigm but combined extracellular stimulation with whole-cell patch clamp recordings of NG2 cells. Synaptic stimulation (100 Hz, 1 s) depolarized cells to almost −30 mV and evoked a small and long lasting increase in the fluorescence of the calcium indicator dye (400 µM Fluo-4) suggesting a recruitment of voltage-gated calcium channels by synaptic input. Both studies analyzed the soma of NG2 cells only and it is tempting to speculate that much weaker synaptic input may elicit local calcium transients in dendrites (see below). The study by Wake et al. (2011), for which as discussed above it is unclear whether synaptic neuron-NG2 transmission is involved, suggests that NG2 cells may possess a signaling apparatus able to generate dendritic calcium signals which do not propagate to the cell body.

Glutamate released synaptically onto NG2 cells could also increase cytosolic calcium concentration independent of membrane depolarization. Pharmacological activation of metabotropic glutamate receptors has been shown to cause pronounced and long-lasting calcium signals (Luyt et al., 2003; Haberlandt et al., 2011). However, to date there is no evidence that synaptic stimulation can activate metabotropic receptors on NG2 cells; this is in striking contrast to astrocytes for which a large number of studies shows that metabotropic glutamate receptors are the prime route to increase intracellular calcium in response to neuronal activity (Verkhratsky et al., 2012). In NG2 cells, calcium entry through calcium-permeable ionotropic glutamate receptors may be another way to initiate synaptic calcium signaling. The participation of calcium-permeable AMPA receptors in synaptic responses has been tested by assessing their voltage-dependence and pharmacological profile. In early postnatal hippocampus these experiments suggest that at least some of the synaptic AMPA receptors lack the GluRB subunit and are calcium-permeable (Bergles et al., 2000; Ge et al., 2006). In corpus callosum, synaptic AMPA receptors are almost completely calcium-impermeable (Kukley et al., 2007; Ziskin et al., 2007), and AMPA receptors only become calcium-permeable at later developmental stages (Ziskin et al., 2007). Calcium elevations in NG2 cells during bath-application of glutamate to hippocampal slices were taken as evidence for calcium entry through AMPA receptors (Ge et al., 2006). However, the results of this study have to be interpreted with caution as glutamate applied to brain slices has many target receptors on non-NG2 cell membranes, and it is difficult to rule out triggering of secondary mechanisms which may also lead to calcium elevations in NG2 cells. At present, there is no direct evidence for calcium entry through AMPA receptors into NG2 cells during synaptic activity. Considering that synaptic currents in NG2 cells are relatively small; there are probably only around 100 synaptic contacts per cell; only a proportion of AMPA receptors are calcium-permeable and the fractional calcium current through these receptors is only about 10% and less than a 10th of total calcium ions entering will appear as free calcium in the cytoplasm, the question arises: is it still reasonable to expect calcium signals in NG2 cells mediated by direct entry through AMPA receptors? To address this question we need to estimate how many calcium ions enter if a single vesicle of glutamate is released onto an NG2 cell. If we use the kinetic properties summarized above and assume that a quantal glutamate current rises linearly to a peak amplitude of 10 pA and then decays back to baseline with a time constant of 1.5 ms the charge transferred amounts to 2 × 10^−14^ C. If we further assume that half of synaptic AMPA receptors are of the calcium-permeable type and that the fractional calcium current through these receptors is 10%, we arrive at a quantal synaptic calcium charge of 1 × 10^−15^ C which translates, with the help of the Faraday constant and the double charge of calcium ions, into roughly 5 × 10^−21^ mol, corresponding to ~3000 calcium ions. If these calcium ions distribute within the soma (diameter 6.5 µm), the total calcium concentration in the soma would increase by approximately 30 nM. However, most calcium ions in the intracellular environment at least 95% (Matthews et al., 2013) will be bound by cellular buffers and therefore the resting free calcium concentration (likely around 100 nM in NG2 cells, Haberlandt et al., 2011) would only negligibly increase by 1.5 nM (30 nM × 5%). But if we consider synchronous activation of 100 neuron-NG2 cell synapses (*P_r_* = 25%) we can indeed expect a detectable increase in the free calcium concentration by up to ~40 nM. Considering that most synaptic contacts will be formed with the thin NG2 cell dendrites it is worth estimating how the local calcium concentration would be changed in a subsynaptic dendritic segment. For simplicity we suppose that the calcium ions entering the dendrite during a quantal glutamatergic event disperse within 50 ms in a 3 µm dendritic segment (diameter 0.2 µm). This dendritic volume is roughly 1000-fold smaller than the soma of the cell, and as a result a single glutamate-filled vesicle could increase the free (not total) calcium concentration by as much as 1–2 µM. Thus, even if much less than 50% of AMPA receptors are calcium-permeable and if synaptic density and synchrony is scarce there should be pronounced dendritic calcium signals coding the activity of nearby axons. NMDA receptors can have an even higher calcium permeability than AMPA receptors (Schneggenburger et al., 1993; Burnashev et al., 1995) but their recruitment by synaptic glutamate release is negligible and their total number appears to be much lower than the number of AMPA receptors (De Biase et al., 2011). Furthermore, the very negative resting membrane potential of NG2 cells supports the inhibition of NMDA receptors by magnesium and a contribution of this type of glutamate receptor to synaptic calcium signaling is therefore much less likely.

### Intracellular sodium, chloride and potassium concentration

Whereas a change in intracellular calcium concentration is readily recognized as an important signaling mechanism, the situation is less evident for sodium, chloride and potassium ions. Still, there is evidence that concentration changes of these ions can also at least indirectly profoundly change intracellular signaling. Sodium ions display an enhancing effect on NMDA receptor activity (Yu and Salter, 1998) and have been shown to inhibit the cell cycle of oligodendrocyte precursor cells presumably via block of potassium channels (Gallo et al., 1996; Knutson et al., 1997; Ghiani et al., 1999; Schroder et al., 2002). Sodium ions were also reported to be coupled to intracellular calcium increases via the entry mode of the sodium-calcium exchanger (Tong et al., 2009). The intracellular chloride concentration, which has been found to be approximately 50 mM in NG2 cells (Passlick et al., 2013) is important for setting the amplitude and direction of GABA-ergic potentials. Furthermore, an inhibitory action of GABA-A receptor activation on AMPA receptor activity has been described which was partially attributed to an action of chloride ions on the AMPA receptor channel (Lin and Bergles, 2004).

As seen above, ion concentration changes are expected to be orders of magnitudes larger in dendrites of NG2 cells due to their much smaller volume. If we use the transferred charge calculated for a quantal glutamatergic event above and, for simplicity, completely attribute it to sodium ions, an increase in dendritic sodium concentration by 2 mM is expected. During temporal summation of repetitive activity of an individual synapse the concentration increase could easily reach tens of millimolar and by this provide a significant shift of the resting intracellular sodium concentration from ~10 mM (Blaustein and Lederer, 1999). Such a shift of the sodium concentration, together with the associated synaptic depolarization, and an elevation of intracellular calcium concentration mediated by some calcium-permeable AMPA receptors would strongly favor the calcium-entry mode of sodium-calcium exchangers and could boost dendritic calcium signaling in NG2 cells (Blaustein and Lederer, 1999). The presence of functional sodium-calcium exchangers and their operation in calcium-entry mode was recently reported in NG2 cells which were bathed in GABA-A receptor agonists (Tong et al., 2009). Note that our above conclusions are valid for dendrites only, as even maximal synaptic input would cause only insignificant changes of the sodium concentration in the cell body due to its thousand-fold larger volume.

Quantal GABA-A receptor mediated synaptic currents display substantively slower kinetics when compared to glutamatergic transmission, and therefore also transfer a substantially larger charge. If we approximate the kinetics of the currents with a linear rise of the chloride-driven current over the first 3 ms and an ensuing exponential decay with a time constant of 30 ms, the total charge transferred in 50 ms is approximately 2.5 × 10^−13^ C, roughly 10-fold larger than that of their glutamatergic counterpart. Since synaptic depolarization by GABA-A receptors corresponds to outward flux of chloride ions the transferred charge predicts that the local intracellular chloride concentration temporarily drops by up to 26 mM. This means a significantly reduced driving force for chloride ions and predicts that the observed paired-pulse depression of IPSCs (Lin and Bergles, 2004) may be partially caused by intracellular chloride depletion. Further, this calculation proposes that in small dendrites of NG2 cells a train of incoming depolarizing GABA-ergic postsynaptic potentials may primarily act in an inhibitory manner through shunting the propagation of glutamatergic input along dendrites.

In our eyes, significant changes in the intracellular potassium concentration are not expected even in dendrites during on-going synaptic activity for two reasons: firstly, the resting membrane potential of NG2 cells is close to the potassium reversal potential such that the driving force for potassium ions usually is very small (in the absence of a long-lasting depolarization). Secondly, the intracellular potassium concentration is high, approximately 150 mM, meaning that only concentration changes in the tens of millimolar range would produce relevant percentage changes in the potassium concentration.

## Possible implications for activity-dependent myelination

What does the mode of synaptic signal integration by NG2 cells tell us about possible functional roles of neuron-NG2 cell synapses in myelination? First of all it is important to recall that direct synaptic transmission provides a high spatial and temporal resolution which is not shared by other neuron-glia signaling pathways. For example, non-vesicular release of ATP into the extracellular space and its subsequent degradation is a slow process which requires several hundred milliseconds before the signaling molecules reach NG2 cells. As a result ATP release cannot precisely encode the timing of action potentials and NG2 cells cannot discriminate which of the neighboring axons has released the molecules because diffusion blurs the origin of ATP. In contrast, direct glutamatergic synaptic transmission happens within a millisecond and the diffusional distance to the NG2 cell is only a few nanometres across the synaptic cleft. The integration properties of NG2 cells determine what NG2 cells can do with this precise information about the activity of individual axons. As outlined above, voltage-gated channels strongly determine which pattern of synaptic activity produces a maximal depolarization of NG2 cells or possibly a calcium increase. For example, NG2 cells may preferentially respond to brief high-frequency trains of synaptic activity, to occasional strong synaptic input, to persistent weak activity or a combination thereof. On the other hand, the passive electrical properties and the subcellular distribution of ion channels and signaling mechanisms are crucial for the ability of NG2 cells to selectively respond to input from individual axons. If synaptically induced depolarizations or calcium signals remain sufficiently local, NG2 cells could more easily generate local actions which affect only the axon which gave rise to the input.

In our eyes synaptic signal integration in NG2 cells could be one of the crucial steps involved in activity-dependent myelination. Most white matter tracts such as corpus callosum contain at least 20–30% unmyelinated axons. As there is good evidence that neuronal activity can increase myelination (Fields, 2008), and possibly the number of myelinated axons, it is likely that the these unmyelinated axons are principally myelinatable. Oligodendrocytes, in contrast to NG2 cells, are not very plastic, do not receive synaptic input (Frohlich et al., 2011) and cannot proliferate and are therefore unlikely to be able to contact and ensheath additional axons. We consider it more likely that NG2 cells respond to synaptic activity and contact and select unmyelinated axons for *de novo* myelination. The signal integration properties of NG2 cells would determine which axon is preferentially selected for new myelination. Currently, there is no solid empirical evidence for which kind(s) of *in vivo* axonal activity may be required to facilitate *de novo* myelination but there are two general scenarios. In one simplistic scenario there is a general and fixed rule according which NG2 cells select axons for myelination, for example if axons fire above a minimally required frequency, e.g., above 50 Hz. The signal integration properties of NG2 cells may in this scenario be tuned to optimally respond to firing frequencies of 50 Hz and higher. However, we consider this scenario to be unlikely because such a general rule of activity dependent myelination does not optimize conduction velocity. Certainly, myelination according to a general rule will speed action potential velocity but would it make sense to increase conduction velocity for all axons firing at high (or low) frequencies? What really matters for a neural circuit is the timing of an action potential; the speed of action potential propagation along a given axon is optimal if the transmitter released from the nerve terminal of this axon onto the postsynaptic neuron arrives at the right time. Whether or not it is the right time is decided by this postsynaptic neuron and depends on the timing relative to other synaptic inputs arriving at this cell. In the end, it is a lack of synchrony between the firing of a given axon and other axons converging on the same postsynaptic neuron which determines the need for myelination and which confers neurophysiological relevance to the process of activity-dependent myelination. In this scenario the postsynaptic neuron must signal retrogradely to the axon and indicate whether or not action potential conduction needs to be accelerated. Indeed, depending on the function of the postsynaptic neuron in its local network the conduction in low or high frequency firing afferent axons may need to be accelerated. How can the axon, which received this retrograde information from its postsynaptic partner, indicate the need for myelination to NG2 cells? Obviously, it cannot just alter its mean firing frequency as this would change the information which is transmitted to the postsynaptic neuron and may not be compatible with the function of the connected neuronal network. Instead, the axon could change the transmitter release machinery at the axon-NG2 cell interface to selectively alter synaptic signaling to NG2 cells. This could lead to either a stronger synaptic response (larger mean amplitude of synaptic input) or to a response with completely different short-term plasticity (e.g., by the varying the pool of release-ready synaptic vesicles). Here again synaptic integration properties of NG2 cells are crucial and may be tuned to detect such axonally induced alterations of synaptic input, such as large synaptic events or a particular temporal pattern of synaptic input (facilitating or depressing train of synaptic potentials).

As mentioned above, regional neuron-NG2 synaptic signaling seems to be most pronounced prior to the first strong wave of myelination, a favorable time to tune myelination according to neuronal activity. Neuron-NG2 synapses are consistently maintained through adulthood. However, synapses on NG2 cells in adult animals likely have a greatly reduced electrical impact because the membrane properties of NG2 cells systematically change with the age of the animal. Membranes become electrically leaky and voltage gated channels are less prominent. Therefore, in the majority of NG2 cells of adult animals synaptic input would not be expected to trigger a cellular action. It will be interesting to see whether those NG2 cells which, infrequently, generate oligodendrocytes in the adult (Rivers et al., 2008; Hughes et al., 2013), regain or maintain a juvenile electrophysiological phenotype. Another option for oligodendrogenesis in the adult CNS is to draw upon presumably juvenile NG2 cells recruited from the subventricular zone: during remyelination in the corpus callosum NG2 cells were shown to migrate to the lesion where they establish synapses with supposedly severed axons (Etxeberria et al., 2010).

## Conclusion

In summary, in the quest for additional functional roles of NG2 cells and for the impact of the enigmatic synaptic input to NG2 cells it is a key step to identify the ways in which the responses generated by classical vesicular release of neurotransmitters from nearby axons are integrated and set into action by NG2 cells. Based on the present comparison of the properties of synaptic input to the properties of voltage gated ion channels expressed by NG2 cells, our simulation of voltage propagation, and estimates of intracellular ion concentration changes, we propose that NG2 cells utilize complex local signal integration and may also respond locally only to specific patterns of presynaptic neuronal activity. However, it should be kept in mind that both our simulation and our concentration estimates follow a fairly simplistic view with the purpose of providing a general picture and a comparison to neuronal signal integration and should not be taken as exact absolute numbers.

## Conflict of interest statement

The authors declare that the research was conducted in the absence of any commercial or financial relationships that could be construed as a potential conflict of interest.
